# Effects of Dietary Paramylon on Nutrient Digestion and Absorption and Intestinal Health of Weaned Piglets

**DOI:** 10.3390/ani16020304

**Published:** 2026-01-19

**Authors:** Tianjiao Wu, Zhiming Zhang, Zheng Luo, Fangbao Shu, Qi Han, Jie Yin, Peng Bin

**Affiliations:** 1 College of Animal Science and Technology, Hunan Agriculture University, Changsha 410128, China; wutianjiao2026@126.com (T.W.); 15890679272@139.com (Z.Z.); 15674506212@163.com (F.S.); binpeng23@scau.edu.cn (P.B.); 2 Key Laboratory of Livestock and Poultry Resources Evaluation and Utilization, Yuelushan Laboratory, Changsha 410128, China; 3 Kemin (China) Technologies Co., Ltd., 25 Qinshi Road, Sanzao, Zhuhai 519040, China; leo.luo@kemin.com; 4 Key Laboratory of Agro-Ecological Processes in Subtropical Region, Institute of Subtropical Agriculture, Chinese Academy of Sciences, Changsha 410125, China; 18404985053@163.com

**Keywords:** Paramylon, β-glucan, weaned piglets, nutrient digestibility, nutrient transportation, gut microbiota

## Abstract

Weaning stress frequently compromises intestinal integrity and nutrient absorption in piglets and induces structural perturbations in the gut microbiota. This study investigated the effects of dietary Paramylon, a linear β-1,3-glucan from *Euglena gracilis*, on growth and intestinal function. A total of 32 healthy, 21-day-old weaned piglets (male, castrated, Duroc × Landrace × Yorkshire) were randomly allocated to four groups: control, 0.025%, 0.05%, and 0.1% Paramylon (8 pigs/group). The results showed that 0.05% dietary Paramylon quadratically increased average daily gain and feed intake (*p* < 0.05). Serum TC content significantly increased, while the serum urea level significantly decreased (*p* < 0.05). This optimal dose was used for mechanistic exploration. Dietary 0.05% Paramylon notably enhanced ileal morphology, increasing the villus height to crypt depth ratio (*p* < 0.01) and significantly improving apparent nutrient digestibility and ileal β-amylase activity (*p* < 0.05). The expression levels of *SLC7A1* and *GLUT2* in ileum tissues were significantly upregulated (*p* < 0.05). The expression level of *SLC7A7* in the liver was also increased (*p* < 0.05). This nutrient transport-promoting effect was further confirmed in IPEC-J2 cells, which manifested because 10 ng/mL of pure Paramylon significantly upregulated the gene expressions of *SLC38A2*, *EAAT3*, *PEPT1*, and *GLUT2* (*p* < 0.05). KEGG enrichment analysis on the ileum indicated that differentially abundant metabolites were enriched in amino acid-related metabolic pathways. Furthermore, the 16s sequencing results revealed that *Romboutsia* was significantly enriched in the Paramylon group. In conclusion, Paramylon, as an effective dietary supplement, helps promote nutrient digestion and absorption in weaned piglets and contributes to maintaining intestinal health.

## Simple Summary

Natural polysaccharides are gaining widespread attention as sustainable and bioactive feed additives. However, the full potential of many novel polysaccharides remains underexplored. This study focused on Paramylon, a unique β-glucan extracted from *Euglena gracilis*, to explore its broad potential as a functional nutrient. We found that dietary Paramylon significantly enhanced the growth and nutrient digestion of weaned piglets. It improved the structure and function of the intestine, upregulating key transporters for amino acids to facilitate nutrient absorption. This core mechanism was confirmed in both animal and cellular models. Our work demonstrated that Paramylon is an effective polysaccharide that can enhance digestive health and nutrient uptake of weaned piglets, providing a scientific basis for its application as a novel, natural additive to support sustainable animal production.

## 1. Introduction

Weaning stress represents a significant challenge in swine production. During the weaning period of piglets, multiple factors typically induce morphology and functional alterations in the small intestine, disrupt the intestinal microbiota balance, and impair digestive and absorptive capacity, ultimately resulting in growth retardation and elevated mortality [1,2]. The gastrointestinal tract is the crucial area for the digestion and absorption of nutrients [3], making intestinal health critical for piglet growth and development. In animal husbandry, the use of antibiotics is subject to increasingly stringent regulations. In many countries and regions, the routine use of antibiotics for growth promotion has been banned or is strictly restricted [4]. However, the prohibition of antibiotics in feed will significantly affect the growth performance of animals, increase mortality rates, and hinder livestock industry development. Therefore, identifying healthy and safe anti-weaning-stress additives to enhance intestinal health and nutrient utilization efficiency in piglets is imperative.

Natural polysaccharides are ubiquitously distributed across diverse biological kingdoms, serving as essential structural components and energy reserves. Among them, β-glucans represent a prominent class of dietary fiber polysaccharides composed of linear polymers of D-glucose subunits interconnected by glycosidic bonds. These polymers are structurally characterized by the presence of three specific glycosidic linkages: β-(1,3)/β-(1,4)/β-(1,6) [5]. β-1,3-glucan is a class of carbohydrates naturally produced by various taxonomic groups, including plants, fungi, bacteria, and select freshwater algae, as a constituent of their cell walls. These glucans are abundantly available and cost-effective [6], while exhibiting notable bioactivities, such as anti-inflammatory, antioxidant, and immunomodulatory properties [7]. It is important to note that β-glucans from different sources exhibit distinct structural features, which underpin their unique physicochemical and biological properties. Paramylon, isolated from the microalga *Euglena gracilis*, is characterized by an exceptionally high degree of polymerization and an almost perfectly linear backbone linked exclusively by β-(1→3) glycosidic bonds [8]. This simple and homogeneous structure markedly contrasts with the mixed-linkage (β-(1→3)/β-(1→4)) pattern typical of cereal β-glucans (e.g., from oats and barley) and the highly branched structures with β-(1→6) side chains found in fungal and yeast β-glucans [9,10]. Emerging evidence indicates that β-1,3-glucan derived from algae can enhance the intestinal health [11] and stress resistance of animals [12,13,14] and exhibit immunomodulatory effects [15,16]. However, studies on the effects of dietary Paramylon on nutrient absorption in weaned piglets remain limited, and the mechanisms involved have not been systematically clarified. This study investigated the effects of dietary Paramylon supplementation on growth performance, nutrient absorption function, and intestinal health in weaned piglets. The findings provide a foundation for developing Paramylon as an eco-friendly functional feed additive and propose potential strategies to address nutrient absorption challenges in weaned piglets.

## 2. Materials and Methods

The animal study was reviewed and approved by the Institutional Animal Care and Use Committee of Hunan Agricultural University (202105).

### 2.1. Materials

The crude Paramylon and pure Paramylon (β-glucan) were supplied by KEMIN Co., Ltd. (Zhuhai, China). The Paramylon (95% pure) was used for the cell experiments.

### 2.2. Animals and Dietary Treatments

Thirty-two Duroc × Landrace × Yorkshire male piglets weaned at 21 days (with a mean body weight of approximately 7.5 kg and of similar body condition) were randomly assigned to four groups (eight piglets per group). All piglets were housed individually as single replicates within a common room. The room contained multiple pens (one piglet per pen). The control group (CTRL) were fed a basal diet, and three treatment groups were provided with the same diet supplemented with 0.025%, 0.05%, and 0.1% crude Paramylon. The basal diet was formulated in strict accordance with the NRC (2012) guidelines to ensure that all nutritional requirements were met as per the established standards (Table 1). Before the experiment began, pure chromium trioxide (Cr_2_O_3_) powder was premixed with the feed raw materials in a stepwise manner according to the addition ratio of 0.2%, serving as an exogenous digestibility indicator. The temperature in the pigsty was controlled at 28–30 °C in the first week and was subsequently reduced by 2 °C on a weekly basis. Relative humidity was maintained within the range of 50–60%. During the 28-day trial, the piglets were fed once at 8:00, 12:00, 18:00, and 22:00 every day. All piglets were housed under conditions that ensured the continuous availability of drinking water. The amount of feed given and remaining feed were recorded daily, and the health conditions of the pigs were observed.

### 2.3. Growth Performance

The daily feed amount and remaining feed amount were accurately recorded for each individual piglet, and each piglet was weighed on the 1st day and 28th day. The average daily feed intake (ADFI), average daily gain (ADG), and feed-to-weight ratio (F/G) were determined to evaluate the growth performance of each group.

### 2.4. Sample Collection

On day 28 of the experiment, blood samples were collected from the anterior vena cava of each weaned piglet following a 12 h fast, using 10 mL vacuum tubes. Samples were placed at 4 °C for 40 min and centrifuged at 845 rcf (g) for 15 min, then stored at −80 °C until analysis. Following anesthesia induced by 3% sodium pentobarbital (25 mg/kg), the piglets were subsequently euthanized via exsanguination. The abdominal cavity was opened for immediate small intestine isolation and sampling. For histomorphological analysis, segments of the ileum were collected, cleansed with physiological saline, and immersion-fixed in 4% paraformaldehyde. Approximately 2 cm sections of ileum tissue were clipped, rinsed with normal saline, and transferred into 2 mL aseptic centrifuge tubes, stored in a liquid nitrogen. And ileal digesta samples in sterile 2 mL tubes were rapidly frozen in liquid nitrogen.

### 2.5. Serum Biochemical Parameter Analysis

The concentrations of serum total protein (TP), urea, albumin (ALB), globulin (GLB), total cholesterol (TC), triglyceride (TG), high-density lipoprotein (HDL-C), and low-density lipoprotein (LDL-C) were measured by Wanwei Biology (Changsha, China).

### 2.6. Free Amino Acid Contents

The contents of free amino acids in serum samples, including lysine (Lys), methionine (Met), isoleucine (Ile), histidine (His), glycine (Gly), threonine (Thr), taurine (Tau), ornithine (Orn), and others, were determined by high-performance liquid chromatography (HPLC). Serum samples were first centrifuged (12,000× *g*, 4 °C, 10 min). Then, 500 μL of supernatant was transferred to a 1.5 mL centrifuge tube and mixed with the same volume of 8% sulfosalicylic acid and placed at 4 °C overnight. The supernatant was recentrifuged under identical conditions and filtered through a microporous membrane (0.22 μm) into the sample bottle with a 1 mL syringe, and the serum amino acid concentrations were determined by high-performance liquid chromatography (Agilent 1290, Agilent Technologies, Santa Clara, CA, USA).

### 2.7. Analysis of Digestibility of Nutrients

Chromium oxide (Cr_2_O_3_) served as an exogenous indicator for the digestion test. 0.3% Cr_2_O_3_ was added to the experimental diet as the indigestible exogenous marker. Feed and fecal samples were collected four times daily during the final 3 experimental days. Daily feed samples (50 g per group per collection) were collected, and feed samples of each group were mixed and stored evenly. Fresh fecal samples (~100 g/pen per collection) were collected and pooled by treatment. Samples were thoroughly mixed and dried in the drying oven (65 °C) for 72 h, then grinded through a 4 mm screen. The dry matter (DM), crude fiber, crude fat and crude protein (CP) of the samples were analyzed. Gross energy (GE) was measured using the Parr 6400 Automatic Oxygen Bomb Calorimeter (Parr Instrument Co., Moline, IL, USA). Chromium concentrations were quantified by graphite furnace atomic absorption spectrometry for digestibility calculations. The calculation formula is as follows: apparent total tract digestibility (ATTD, %) = 100 − [100 × (C1 × F2)/(C2 × F1)], where C1 and F1 represent Cr_2_O_3_ and nutrient content (%) in the feed, respectively, and C2 and F2 represent Cr_2_O_3_ and nutrient content (%) in the feces, respectively.

### 2.8. Intestinal Morphology

Following fixation in 4% paraformaldehyde, the tissue sections were processed through a standard series for histological examination, including deparaffinization, rehydration, and hematoxylin and eosin staining. Morphological examination was performed using a Carl Zeiss imaging system (Carl Zeiss, Oberkochen, Germany). The villus height/crypt depth (V/C) ratio was quantified by evaluating at least six randomly selected fields from each tissue section with CaseViewer software (Version 2.3).

### 2.9. Measurement of Enzyme Activities

The activities of digestive enzymes in the ileal digesta (trypsin, β-amylase, and lipase) were measured via assay kits purchased from Beijing Boxbio Science & Technology Co., Ltd. (Beijing, China). All steps for detection were performed according to the instructions from the manufacturer.

### 2.10. Quantitative Real-Time PCR Analysis

Ileal samples were homogenized in liquid nitrogen and total RNAs were extracted using TRIzol reagent (Sigma, Saint Louis, MO, USA). Complementary DNA (cDNA) was synthesized from the RNA samples using the PrimeScript™ RT Reagent Kit with gDNA Eraser (TaKaRa, Dalian, China). The subsequent fluorescence-based quantification step was performed utilizing TB Green^®^ Fast qPCR Mix on a LightCycler 480 System II (Roche, Shanghai, China). The gene expression was calculated using the method of 2^−ΔΔCt^ [17]. Table 2 lists the primer sequences employed in this study.

### 2.11. IPEC-J2 Cell Culture

The porcine jejunum epithelial cell line IPEC-J2 used in this experiment was cryopreserved in liquid nitrogen by our research group. IPEC-J2 cells were cultured in DMEM/F12 medium containing 10% fetal bovine serum (FBS, Gibco, Thermo Fisher Scientific, Grand Island, NE, USA) and 1% double antibody (Gibco) in a carbon dioxide incubator at 37 °C, 5% CO_2_, and 95% humidity. Cells were maintained with a change in medium every 48 h and were passaged or harvested prior to exceeding 80% confluence. The culture medium was removed, and the cells were washed twice with PBS. A total of 1 mL of pancreatin containing EDTA was taken and digested at 37 °C for 4 min. Then digestion was terminated with 2 mL of culture medium. Following collection, the cells were centrifuged at 1000× *g* for 5 min. After supernatant removal, the resulting pellet was finally re-suspended in complete medium for passage or planking. Purified Paramylon (95% purity) was used in the cell experiments.

### 2.12. CCK8 Assay

The proliferation of IPEC-J2 cells was examined using a CCK-8 Assay Kit (DOJINDO, CK04, Mashiki, Japan). The IPEC-J2 cells were cultured in 96-well plates and cultured for 24 h. Paramylon was prepared in DMSO at 40 mg/mL. Subsequent dilution to the desired working concentrations was carried out using the cell culture medium. Cells were treated with a wide range of Paramylon concentrations (0 ng/mL, 0.1 ng/mL, 1 ng/mL, 10 ng/mL, 25 ng/mL, 50 ng/mL, and 100 ng/mL) for 24 h. A control group containing 0.25% DMSO was included. Cultures were supplemented with 10 μL of CCK-8 solution per well and subsequently incubated for 2 h. The OD values of each hole at a 450 nm wavelength were detected by enzyme labeler (TECAN, Männedorf, Switzerland).

### 2.13. Untargeted Metabolomics

Ileum samples of 40 mg were retrieved from −80 °C grinding beads and 400 μL extract (methanol/water = 4:1 (v:v), containing 0.02 mg/mL L-2-chlorophenylalanine), respectively. The sample solutions were placed in a frozen tissue grinder for 6 min (−10 °C, 50 Hz) and then extracted by ultrasound at low temperature for 30 min (5 °C, 40 kHz). After standing at −20 °C for 30 min and centrifuging at 13,000× *g* for 15 min (4 °C), the supernatants were transferred into the injection vial with internal intubation for LC-MS/MS analysis. Each test sample was mixed with equal volume solution to prepare quality control samples (QC). A QC sample was inserted after every 5–15 experimental samples to investigate the repeatability of the entire analysis process. The samples were analyzed by LC-MS/MS using Thermo Field’s ultra-high-performance liquid chromatography tandem Fourier Transform mass spectrometry UHPLC-Q Exactive HF-X system (Shanghai Meij Biomedical Technology Co., Ltd., Shanghai, China).

Separation of the 3 μL samples was achieved using an HSS T3 column (100 mm × 2.1 mm i.d., 1.8 µm) prior to mass spectrometric detection. The mobile phases were composed of (A) 95:5 (*v*/*v*) water/acetonitrile with 0.1% formic acid and (B) a mixture of 47.5% acetonitrile, 47.5% isopropyl alcohol, and 5% water, also containing 0.1% formic acid. The method employed a constant flow rate of 0.40 mL/min and the column was maintained at 40 °C.

The positive ion scanning mode and the negative ion scanning mode were adopted to collect the mass spectrometry signals of the samples, with a mass scanning range of 70–1050 *m*/*z*. The mass spectrometer was operated with sheath and auxiliary gas flows configured to 50 psi and 13 psi, respectively. The auxiliary gas heater was maintained at 425 °C, and the ion transfer tube temperature was set to 325 °C. Spray voltages were applied at ±3500 V for positive and negative ionization modes. A normalized collision energy scheme of 20, 40, and 60 V was employed in cyclical steps. Mass spectrometric analysis was conducted with the primary and secondary resolution configured to 60,000 and 7500, respectively, utilizing data-dependent acquisition for spectral collection.

### 2.14. Microbial Analysis

Total DNA was extracted from the ileal contents of piglets using the TGuide S96 Magnetic Universal DNA Kit (Tiangen, Beijing, China, DP812). The DNA concentration was quantified using a microplate reader (Synergy HTX, GeneCompany Limited, Hong Kong, China) with the 1X dsDNA HS Assay Kit (Yeasen Biotechnology, Shanghai, China). The V3–V4 region of bacterial 16S rRNA was amplified using primers 338F (5′-ACTCCTACGGGAGGCAGCA-3′) and 806R (5′-GGACTACHVGGGTWTCTAAT-3′). Amplification was performed on the BL1000 automated PCR system (Revvity Co., Ltd., Waltham, MA, USA). Amplicons were purified using VAHTSTM DNA Clean Beads, quantified by 1.8% agarose gel (Beijing Bomei Fuxin Technology Co., Ltd., Beijing, China) electrophoresis, and pooled equimolarly. To sequence the prepared libraries, we employed the Illumina NovaSeq 6000 platform with a 250 bp paired-end sequencing strategy. QIIME2 (https://qiime2.org) was used for analyzing alpha diversity and beta diversity.

### 2.15. Statistical Analysis

All data were expressed as the mean with standard error of the mean (SEM). Statistical analyses were performed using SPSS (version 26.0). Growth performance and serum biochemical indicators were subjected to one-way ANOVA, and the Duncan’s post hoc test was employed for multiple comparisons. The impact of Paramylon supplementation concentration was evaluated via linear and quadratic orthogonal polynomial models. Other indicators were compared using Student’s t-test. A *p*-value < 0.05 was considered statistically significant, and values between 0.05 and 0.10 were considered indicative of a trend. For graphical representations, GraphPad Prism 9.0.0 was used, with significance levels denoted as * *p* < 0.05, ** *p* < 0.01, and *** *p* < 0.001.

## 3. Results

### 3.1. Effects of Paramylon on Growth Performance in Weaned Piglets

As shown in Table 3, compared with the control group, dietary supplementation of 0.05% Paramylon significantly increased the average feed intake of weaning piglets (*p* < 0.05) but had no significant effect on the daily gain and ratio of feed to gain (*p* > 0.05). Notably, the growth response to increasing dietary levels of Paramylon exhibited significant quadratic effects. This was evidenced by the changes in both ADFI (*P*-Quadratic = 0.030) and ADG (*P*-Quadratic = 0.038). The values for these parameters increased to a peak at the 0.05% inclusion level and declined at the 0.10% level.

### 3.2. Effects of Paramylon on Serum Biochemical Parameters in Weaned Piglets

The results showed that serum TC was significantly increased and serum urea was significantly decreased in the 0.05% Paramylon group (*p* < 0.05). However, there were no statistically significant differences in TG, LDL-C, TP, ALB, GLB, and A/G levels among the groups (*p* > 0.05) (Table 4).

### 3.3. Effects of Paramylon on Serum Free Amino Acid Concentrations in Weaned Piglets

According to the results in Table 5, the research indicated that adding 0.05% Paramylon to the diet has a significant increasing effect on the free amino acid Car and significant decreasing effect on β-Ala in the serum of weaning piglets (*p* < 0.05). However, it has no significant effect on other serum free amino acids, including P-Ser, Tau, PEA, Urea, Asp, Thr, Ser, Glu, α-AAA, Gly, Ala, Cit, α-ABA, Val, Cys, Met, Cysthi, Ile, Leu, Tyr, Phe, Hylys, Orn, Lys, 1Mehis, His, 3Methis, and Arg (*p* > 0.05).

### 3.4. Effects of Paramylon on Nutrient Digestibility in Weaned Piglets

As delineated in Table 6, dietary supplementation with 0.05% Paramylon significantly enhanced total nutrient digestibility in weaned piglets (*p* < 0.05). Compared to the control cohort, the experimental group exhibited marked improvements in crude protein digestibility (*p* < 0.05), with concurrent significant increases in dry matter, ether extract, crude fiber, and gross energy utilization (*p* ≤ 0.001).

### 3.5. Effects of Paramylon on Intestinal Morphology in Weaned Piglets

**Figure 1:**
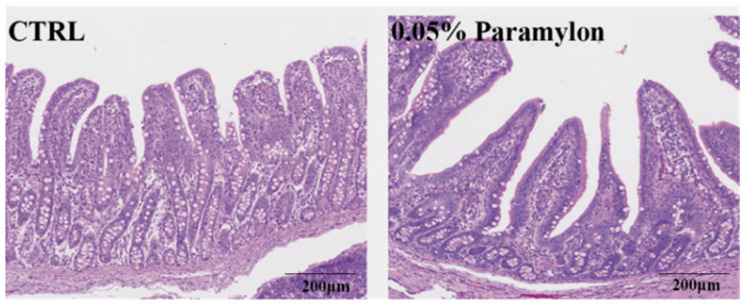
Effects of Paramylon on intestinal morphology in the jejunum. CTRL: basal diet, 0.05% Paramylon: basal diet supplemented with 0.05% Paramylon.

Intestinal morphology was assessed through hematoxylin and eosin (HE) staining of ileal tissue segments. As shown in Figure 1 and Table 7, 0.05% Paramylon treatment significantly increased intestinal villus height and significantly decreased intestinal crypt depth (*p* < 0.01), thereby increasing the V/C (*p* < 0.001).

### 3.6. Effects of Paramylon on Digestive Enzyme Activities in Weaned Piglets

To investigate the effect of Paramylon on digestive enzymes in the ileum of piglets, we measured the activities of TRY, LPS, and β-AL. The results demonstrated that the β-AL activity in the ileal contents of the treatment group was significantly higher than that of the control group (*p* < 0.05), whereas there were no significant differences in TRY and LPS activities (*p* > 0.05) (Table 8).

### 3.7. Effects of Paramylon on Gene Expression of Intestinal Nutrient Transporters in Weaned Piglets

**Figure 2:**
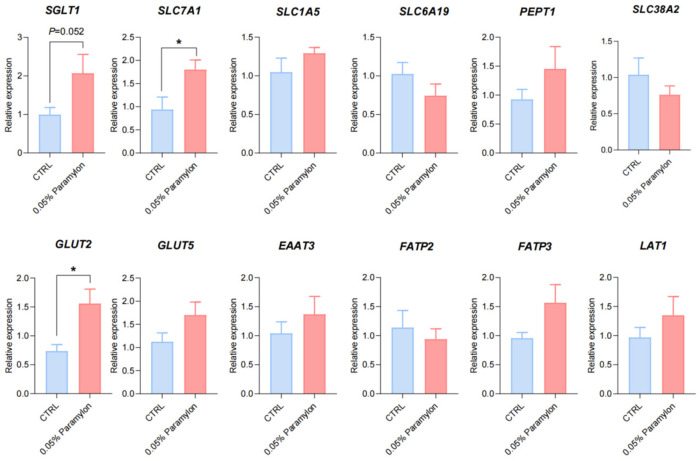
Effects of Paramylon on intestinal nutrient transport. SGLT1: Sodium-Glucose Cotransporter 1; SLC7A1: Solute Carrier Family 7 Member 1; SLC1A5: Solute Carrier Family 1 Member 5; SLC6A19: Solute Carrier Family 6 Member 19; PEPT1: Peptide Transporter 1; SLC38A2: Solute Carrier Family 38 Member 2; GLUT2: Glucose Transporter 2; GLUT5: Glucose Transporter 2; EAAT3: Excitatory Amino Acid Transporter 3; FATP2: Fatty Acid Transport Protein 2; FATP3: Fatty Acid Transport Protein 3; LAT1: L-type Amino Acid Transporter 1. CTRL: basal diet, 0.05% Paramylon: basal diet supplemented with 0.05% Paramylon. Data are presented as means, with error bars representing standard errors. Differences were set to * *p* < 0.05.

The expression of amino acid transporter genes, glucose transporter genes, fatty acid transporter genes, and small peptide transporter genes were detected by extracting ileal RNA, and the effects of dietary 0.05% Paramylon on intestinal nutrient transport of weaning piglets were evaluated at the molecular level. The results revealed that dietary 0.05% Paramylon significantly upregulated the expression of *SLC7A1* and *GLUT2* in the ileal tissue of weaning piglets (*p* < 0.05). *SGLT1* expression showed an increasing trend in the Paramylon group (*p* = 0.052). There were no significant differences in other transporter genes (*p* > 0.05) (Figure 2).

### 3.8. Effects of Paramylon on Hepatic Amino Acid Transporter Expression in Weaned Piglets

**Figure 3:**
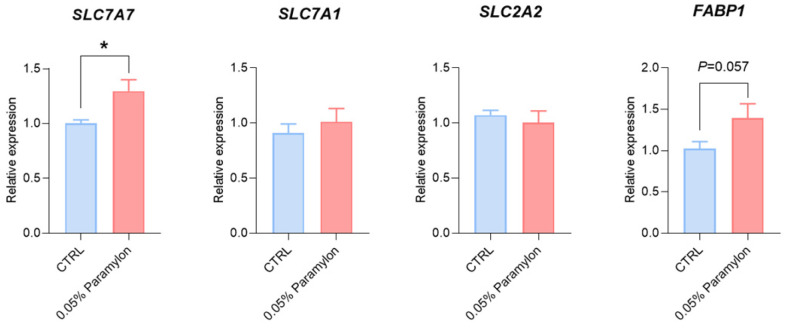
Effects of Paramylon on genes related to nutrient transport in the liver. SLC7A7: Solute Carrier Family 7 Member 7; SLC7A1: Solute Carrier Family 7 Member 1; SLC2A2: Solute Carrier Family 2 Member 2; FABP1: Fatty Acid-Binding Protein 1. CTRL: basal diet, 0.05% Paramylon: basal diet supplemented with 0.05% Paramylon. Data are presented as means, with error bars representing standard errors. Differences were set to * *p* < 0.05.

In this study, the expression level of *SLC7A7* in the liver was significantly increased by 0.05% of Paramylon (*p* < 0.05), while the expression levels of *SLC7A7*, *SLC2A2*, and *FABP1* showed no significant change (Figure 3).

### 3.9. Effects of Paramylon on Proliferation of IPEC-J2 Cells

**Figure 4:**
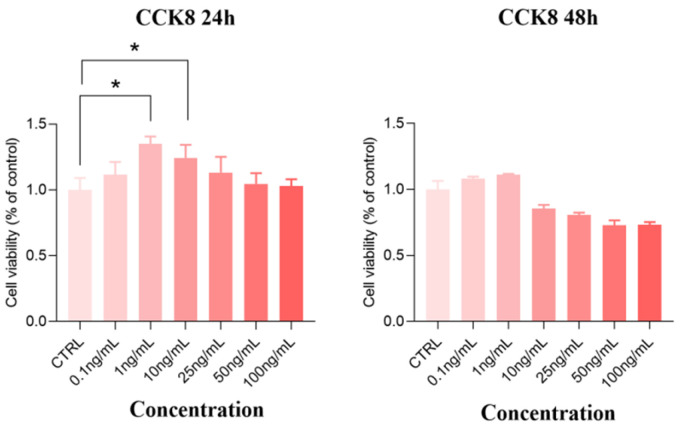
Effects of Paramylon on proliferation of IPEC-J2 cells. CTRL: basal diet, 0.05% Paramylon: basal diet supplemented with 0.05% Paramylon. Data are presented as means, with error bars representing standard errors. Differences were set to * *p* < 0.05.

The IPEC-J2 cells were used to examine the effect of Paramylon on nutrient absorption in vitro. The results of CCK8 showed that both 1 ng/mL and 10 ng/mL Paramylon could significantly improve the cell survival rate after 24 h of treatment (*p* < 0.05), while other concentrations had no significant effect (*p* > 0.05) (Figure 4). And it was observed that there was no significant effect on the cells treated with Paramylon of each concentration for 48 h (*p* > 0.05). Therefore, Paramylon of 1 ng/mL and 10 ng/mL were finally selected for follow-up experiments.

### 3.10. Effects of Paramylon on Nutrient Transporter Genes in IPEC-J2 Cells

**Figure 5:**
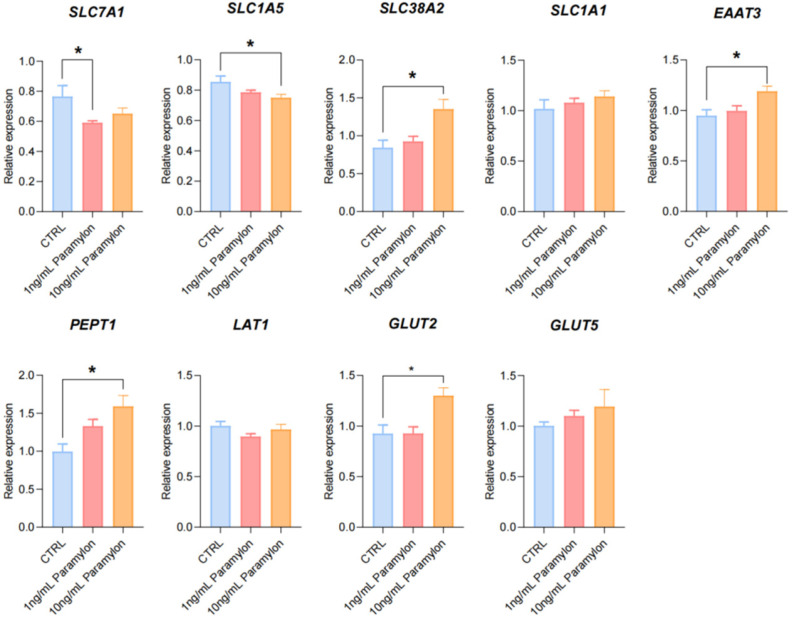
Effects of Paramylon on nutrient transporter genes in IPEC-J2 cells. Data are presented as means, with error bars representing standard errors. Differences were set to * *p* < 0.05.

The impact of Paramylon on IPEC-J2 cells was evaluated at the molecular level by detecting the expression of genes related to nutrient transport and absorption. The results showed that compared with the control group, 1 ng/mL Paramylon treatment significantly downregulated the expression level of *SLC7A1* (*p* < 0.05) while it had no significant effect on the expression of other genes (*p* > 0.05). And 10 ng/mL Paramylon treatment significantly upregulated the expression level of *SLC38A2*, *EAAT3*, *PEPT1,* and *GLUT2* (*p* < 0.05) but it had no significant effect on the expression of other genes (*p* > 0.05) (Figure 5).

### 3.11. Effects of Paramylon on the Ileal Metabolome of Weaned Piglets

**Figure 6:**
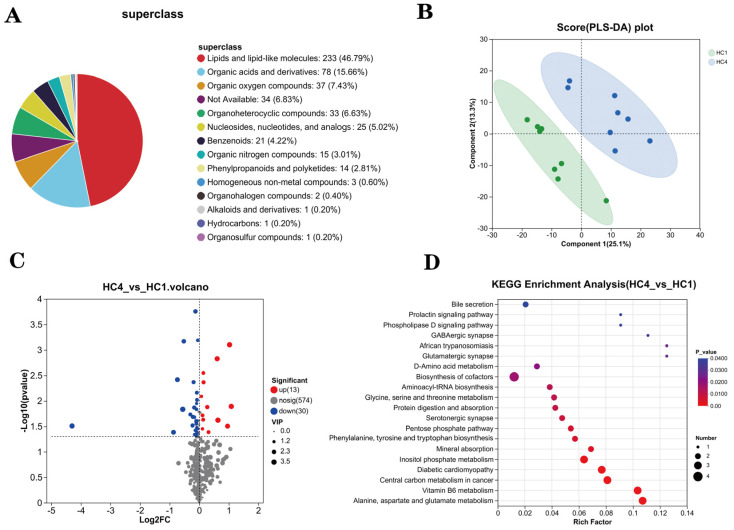
Effects of Paramylon on ileal metabolites (*n* = 8 biologically independent piglets per group). (**A**) Distribution of metabolite superclasses identified in non-targeted metabolomic analysis. (**B**) Orthogonal Projections to Latent Structures Discriminant Analysis (OPLS-DA) score plot. Each point represents an individual biological sample. Green and blue dots correspond to samples from the control and Paramylon-treated groups, respectively. (**C**) Volcano plot of metabolomic differential analysis. Dots are colored/coded by significance: red (significant, *p* < 0.05 and VIP > 1), gray (nonsignificant), and blue (VIP—identified but not significant by *p*-value). (**D**) Bubble plot of KEGG enrichment analysis in metabolomics. HC1: basal diet, HC4: basal diet supplemented with 0.05% Paramylon.

Through non-targeted metabolomic analysis of metabolites in ileum samples of two groups of piglets, a total of 498 metabolites were identified. The statistical classification of 498 metabolites is shown in Figure 6A. The metabolite composition comprised lipids and lipid-like molecules (46.79%), organic acids and derivatives (15.66%), organic oxygen compounds (7.43%), organoheterocyclic compounds (6.63%), nucleosides/nucleotides/analogs (5.02%), benzenoids (4.22%), organic nitrogen compounds (3.01%), and phenylpropanoids/polyketides (2.81%). To validate model quality, permutation tests were utilized to evaluate the OPLS-DA model for overfitting, with the R^2^ and Q^2^ values reporting the fit quality. OPLS-DA revealed significant differences in the abundances of metabolites between two groups (Figure 6B).

A volcano plot indicated that Paramylon supplementation significantly increased the abundances of 13 differential metabolites and decreased the abundances of 30 differential metabolites (Figure 6C). KEGG pathway enrichment analysis was conducted to identify significantly altered metabolic pathways. The analysis of 43 differential metabolites was visualized in a bubble plot (Figure 6D). Differential metabolites between the two groups were mainly concentrated in bile secretion, the phospholipase D signaling pathway, the prolactin signaling pathway, the GABAergic synapse, and the glutamatergic synapse. Notably, differential metabolites were enriched into metabolic pathways associated with amino acids such as D-Amino acid metabolism; phenylalanine, tyrosine, and tryptophan biosynthesis; alanine, aspartate, and glutamate metabolism.

### 3.12. Effects of Paramylon on the Intestinal Bacterial Composition of Weaned Piglets

**Figure 7:**
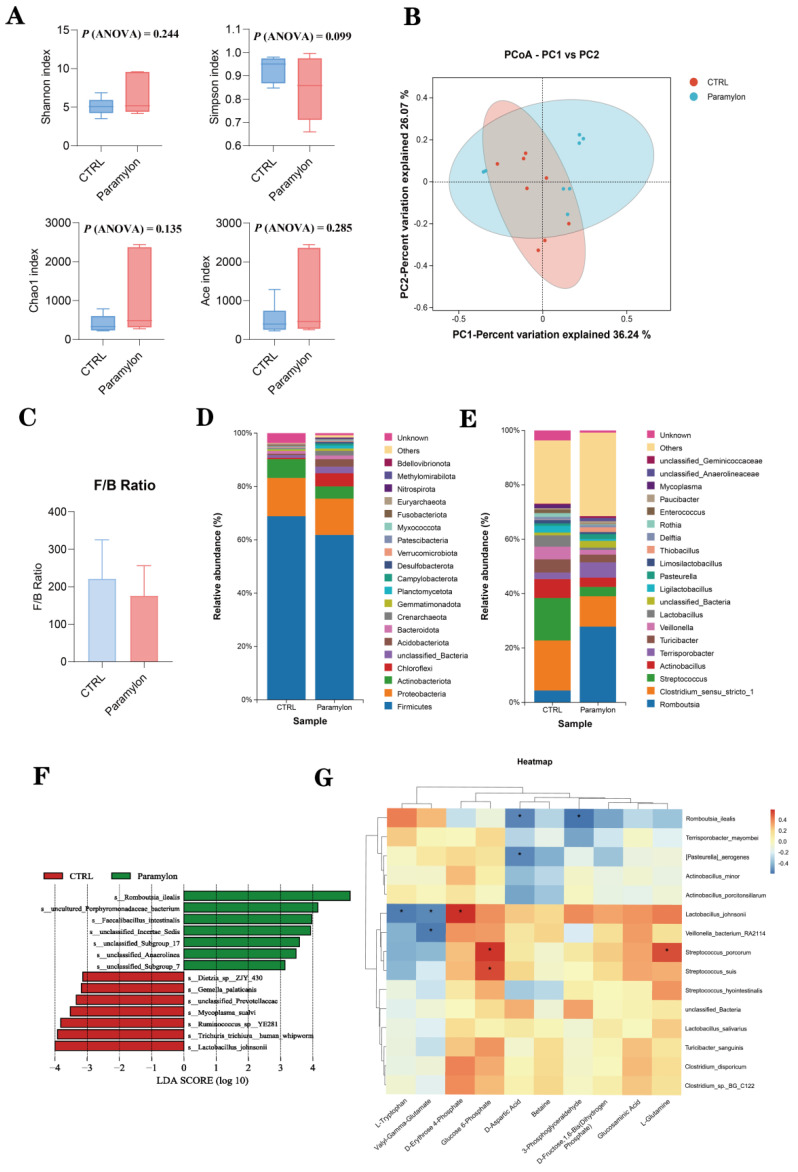
Effects of Paramylon on microbial diversity in ileum of piglets (n = 8 biologically independent piglets per group). (**A**) Shannon index, Simpson index, Ace index, and Chao1 index. (**B**) Principal coordinate analysis (PCoA) plot of bacterial communities based on braycurti. (**C**) Firmicute/Bacteroidete ratio. (**D**) Phyla in ileal contents. (**E**) Genera in ileal contents. (**F**) Species in ileal contents. (**G**) Correlation coefficients between microbial species and metabolites. Red indicates positive correlation; blue indicates negative correlation. HC1: basal diet, HC4: basal diet supplemented with 0.05% Paramylon. Differences were set to * *p* < 0.05.

The α-diversity was assessed using Shannon, Simpson, Chao1, and Ace indices. However, no statistically significant differences were observed in the α-diversity of the Paramylon group compared to the control (*p* > 0.05) (Figure 7A). However, principal coordinate analysis (PCoA) revealed a significant separation in the gut microbiota composition between weaned piglets supplemented with Paramylon and those in the control group (Figure 7B).

Furthermore, the composition of the phylum, genus, and species levels of the ileal microbial community in weaned piglets was analyzed, and the top ten microorganisms at the phylum level and genus level were counted separately (Table 9 and Table 10). No significant differences were detected in the proportions of Firmicutes and Bacteroidetes, and no significant difference in the Firmicute/Bacteroidete (F/B) ratio was observed between the Paramylon group and the control group (*p* > 0.05). At the genus level, supplementation with Paramylon significantly increased the relative abundance of *Romboutsia* and significantly decreased the relative abundance of Lactobacillus (*p* < 0.05).

Figure 7F shows a bar plot of Linear Discriminant Analysis (LDA) effect size (LefSe) analysis, illustrating the significantly different microbial taxa between the CTRL group and Paramylon group. The results showed that *Romboutsia_ilealis* and *Faecalibacillus_intestinalis* were significantly enriched in the Paramylon group.

### 3.13. Correlation Analysis of the Gut Microbiome and Metabolites

Alterations in gut microbiota composition inevitably induce corresponding changes in intestinal metabolites. To identify specific bacterial populations within the chyme that influence metabolic profiles, we performed correlation analyses between the ileal microbiota and detected metabolites. Our study conducted a correlation analysis between the 15 most abundant bacterial species at the species level and ten key metabolites related to the transport and absorption of nutrients (Figure 7G). The results indicated that *Romboutsia_ilealis*, *Pasteurella_aerogenes*, and *Veillonella_bacterium_RA2114* were significantly negatively correlated with the metabolites related to intestinal nutrient digestion and absorption. *Streptococcus_porcorum* and *Streptococcus_suis* showed a significant positive correlation with the related metabolites (*p* < 0.05). Notably, the intestinal bacteria *Lactobacillus_johnsonii* had a significant negative correlation with the metabolites L-Tryptophan and Valyl-Gamma-Glutamate, while it had a significant positive correlation with D-Aspartic Acid (*p* < 0.05).

## 4. Discussion

From neonatal stage to market weight, pigs undergo sequential physiological processes involving nutrient digestion, absorption, and deposition [18]. Post-weaning stress induces significant reductions in feed intake and leads to malnutrition in piglets [19], rendering intestinal nutrient absorption (including proteins, carbohydrates, and lipids) critical for their growth and development during this transitional phase. Natural polysaccharides have demonstrated significant efficacy in enhancing immunity, exerting anti-inflammatory effects, and regulating energy metabolism [20,21]. Notably, various natural polysaccharides have been widely used as feed additives to alleviate weaning stress and enhance growth performance in piglets. Research has confirmed that laminarin polysaccharide supplementation in the diet markedly improves productive performance in weaned piglets [22,23]. Paramylon β-1, 3-glucan is rich in 59 kinds of nutrients essential for animals, including vitamins, mineral nutrients, amino acids, carotenoids, and unsaturated fatty acids. Previous studies have demonstrated β-1, 3-glucan’s potential to enhance animal growth performance and promote overall health [24,25]. Our study revealed a significant quadratic dose–response relationship under restricted feeding conditions, wherein dietary supplementation with 0.05% Paramylon—the identified optimal level—significantly enhanced average daily feed intake and consequently improved the growth performance of weaned piglets. It should be noted that the present study was designed with a focus on elucidating the initial mechanistic responses to Paramylon supplementation. Consequently, systematic clinical assessments, such as diarrhea incidence, were not included as primary endpoints. Future trials aimed at directly evaluating production efficacy should incorporate these essential health indicators.

Dietary macronutrients (carbohydrates, proteins, and lipids) are enzymatically digested and subsequently absorbed across the intestinal epithelium into circulation [26]. Blood biochemical parameters serve as valuable biomarkers for assessing growth performance and metabolic health in animals [27]. The results showed that under restricted feeding conditions, dietary 0.05% Paramylon significantly increased the HDL-C levels and decreased the urea levels. The urea content in the serum is related to protein metabolism and utilization, while the HDL-C content is closely associated with lipid metabolism [28]. This effect may be attributed to dietary Paramylon supplementation enhancing protein utilization efficiency and promoting favorable lipid metabolism in weaned piglets. This interpretation is supported by the established correlation between serum amino acid profiles and animal health status as well as growth performance [29]. The ileum plays a dual role in nutrient assimilation, facilitating the final breakdown of food proteins and serving as the main uptake site for small peptides and amino acids. Therefore, the absorption process of essential amino acids in the small intestine directly influences their serum concentration, whereby enhanced absorption leads to proportionally higher circulating levels [30]. A previous study revealed that dietary supplementation with *Astragalus* polysaccharide could increase the concentration of most essential amino acids in the serum of weaned piglets [31]. However, in the serum of the group with 0.05% Paramylon added, only the concentration of Car significantly increased, while the concentrations of other amino acids did not show any significant changes. The difference may be influenced by the varying fat content in different types of polysaccharides.

Nutrient digestion and absorption, which occur predominantly in the intestine [32], are closely linked to the morphology of the intestinal villi, a structure that plays a pivotal role in this process. The small intestinal morphology of piglets, including VH, CD, and their ratios, are established indicators of intestinal health in piglets [33]. Supplementing the maternal diet with algal polysaccharides has been found to improve intestinal development in offspring piglets, manifested by enhanced overall intestinal morphology and increased villus height in both the jejunum and ileum [34]. Similarly, β-glucan from microalgae has been demonstrated to significantly increase duodenal villus height in nursery pigs [35]. Consistent with this mechanism, our study focused on the ileum under restricted feeding and found that under restricted feeding conditions, 0.05% dietary Paramylon supplementation significantly increased the VH of the ileum in piglets, reduced the CD, and improved the intestinal morphology of the ileum. Enhanced nutrient digestibility may contribute to improved growth performance [36]. While the beneficial effects of β-glucan on nutrient digestibility are well-established in growing-finishing pigs [37], our results demonstrated that this effect extends to weaned piglets, a finding consistent with previous reports [38]. Specifically, 0.05% dietary Paramylon significantly increased total apparent nutrient digestibility compared to the control. This observation reinforces the broader premise that β-glucan supplementation can enhance digestive efficiency across different swine production stages. Improved intestinal morphology, especially increased VH, probably enhances nutrient absorption efficiency [39]. Villus elongation expands the intestinal absorptive surface area, facilitating greater nutrient digestibility and absorption [40]. However, Metzler-Zebeli and Zebeli reported reduced nutrient digestibility with cereal β-glucan supplementation, conflicting with our results [41]. This discrepancy may stem from structural heterogeneity (e.g., β-(1,3/1,6)- and β-(1,3/1,4)-glucan linkages) and compositional divergence among β-glucans derived from distinct biological origins, potentially modulating their biofunctional efficacy in physiological systems [42]. Divergent findings may result from multiple factors including genetic background and environmental conditions.

Post-weaning piglets experience significant stress due to immature digestive function coupled with abrupt dietary and environmental changes. This stress may lead to reduced feed intake, impaired nutrient absorption, and inadequate digestive enzyme secretion, including trypsin, pancreatic lipase, and amylase [43]. Small intestine-derived digestive enzymes catalyze and regulate nutrient digestion [44]. Try, as the predominant proteolytic enzyme in the intestine, serves as a key indicator of protein digestive capacity [45]. The activity of LPS determines the utilization efficiency of fat [46]. These digestive enzymes modulate piglet growth performance by enhancing feed digestion [47]. The results demonstrated that under restricted feeding conditions, dietary supplementation with 0.05% Paramylon significantly increased β-AL activity in the ileal contents of piglets. In contrast, no significant effects were observed on TRY and LPS activities.

Intestinal nutrient absorption is mediated by specialized transport carriers [48], including amino acid transporters, small peptide transporters, and fatty acid and glucose transporters. Slc7a1, a solute carrier family member, facilitates cationic amino acid transport. Dietary starch is enzymatically hydrolyzed to monosaccharides (~80% glucose) that are transported across intestinal mucosa [49]. Monosaccharides bind *SGLT1* at brush-border membranes for active uptake by enterocytes. Upon reaching a threshold intracellular concentration, these monosaccharides subsequently efflux through *GLUT2* via facilitated diffusion along their concentration gradient into the paracellular space [50]. A study has shown that polysaccharides extracted from seaweed can enhance intestinal glucose uptake in swines [51]. Similarly, we found that under restricted feeding conditions, 0.05% Paramylon significantly increased the expression of *SLC7A1* and *GLUT2* in the ileum tissues of piglets. Previous research results have suggested that laminarin-rich extracts can increase nutrient transporter expression in multiple intestinal segments of weaned piglets [52]. Therefore, additional investigation is required to elucidate the function of Paramylon in stimulating nutrient uptake across various segments of the porcine intestine. In this study, the downregulated expression of *SLC6A19*, *SLC38A2*, and *FATP2* could be interpreted as a regulatory response to the specific dietary amino acid profile or flux. Paramylon also significantly increased the expression level of *SLC7A7* in the liver. Notably, the coordinated changes observed in both the ileum and the liver suggest a potential role of the gut–liver axis in mediating the systemic effects of Paramylon. The liver plays a pivotal role in orchestrating systemic nutrient homeostasis through the precise regulation of nutrient transporter expression. The gut–liver axis, a critical functional network linking the intestine and liver through the portal circulation, neuroregulatory pathways, and immunometabolic signaling cascades, serves as a central hub in orchestrating systemic metabolic homeostasis and adaptive responses [53]. The observed upregulation of hepatic *SLC7A7* occurred alongside changes in ileal nutrient transporters (e.g., *SLC7A1* and *GLUT2*), suggesting the possibility of coordinated systemic adaptation. We postulate that Paramylon-induced alterations in intestinal nutrient flux may trigger enterohepatic signaling via the portal circulation, leading to the adaptive hepatic *SLC7A7* upregulation to meet altered metabolic demand. This integrated view posits that Paramylon’s effects extend beyond the gut to systemic metabolic coordination via inter-organ crosstalk. However, it is important to acknowledge a key limitation in interpreting these transcriptional findings. The present study evaluated the expression of key ileal nutrient transporters only at the mRNA level. While these data provide valuable preliminary evidence for potential changes in transport capacity, the functional impact ultimately depends on protein abundance, membrane localization, and activity. Therefore, the mechanistic implications drawn from these transcriptional changes await definitive establishment by corroborating protein-level evidence in the future. The promoting effect of Paramylon on nutrient transport and absorption was validated using IPEC-J2 cells. The cell model recapitulates the intestinal epithelium while excluding the complex milieu of intact tissue, thereby enabling a focused assessment [54]. The research results showed that 1 ng/mL of Paramylon significantly reduced the expression of *SLC7A1*, while 10 ng/mL of Paramylon significantly downregulated the expression of Slc1a5 but upregulated the expressions of *SLC38A2*, *EAAT3*, *PEPT1*, and *GLUT2*.

Non-targeted metabolomic analysis of the ileal tissue revealed that the Paramylon significantly altered the metabolic profiles of lipid and lipoid molecules in the ileum of weaned piglets. The metabolomic analysis revealed a significant enrichment of the identified differential metabolites in biosynthetic and catabolic pathways related to amino acids (e.g., tyrosine, tryptophan, and alanine). This aligned with the observed upregulation of ileal amino acid transporter genes related to amino acid transport in the ileum, suggesting that Paramylon may modulate lipid metabolism and amino acid utilization efficiency, thereby indirectly influencing the transportation of nutrients. Additionally, metabolites were enriched in the protein digestion and absorption pathways. This indicates that Paramylon may indirectly promote protein absorption by influencing the expression of intestinal *PEPT1* rather than directly enhancing the activity of TRY [55]. The enrichment of the bile secretion pathway potentially improved fat digestion via bile acid-dependent lipid emulsification, corroborating our nutrient digestibility results.

The gut microbiota plays a crucial role in maintaining intestinal morphology, facilitating nutrient absorption, and participating in metabolic processes [56,57]. Accumulating evidence suggests that certain natural polysaccharides may exert beneficial health effects through modulation of the gut microbial composition [58]. Dietary β-glucan from *Agrobacterium* sp. has been shown to exert an ileal-specific effect by significantly enriching beneficial microbial communities in weaned pigs. Similarly, the 16s rDNA sequencing results of the ileal chyme of piglets in this study indicated that there were differences in the microbial composition between the group with Paramylon addition and the control group. Previous studies have shown that the F/B ratio is usually associated with certain diseases and intestinal flora disorders [59]. However, at the phylum level, Paramylon showed no significant effect on the F/B ratio in the intestinal microbiota. This could be because the effects of Paramylon are more targeted, influencing specific microbial functions or host–microbe interactions relevant to nutrient metabolism. At the species level, Paramylon significantly increased the enrichment of *Romboutsia_ilealis* and *Faecalibacillus_intestinalis*. These two types of bacteria are both beneficial to the production of SCFAs such as acetic acid and butyric acid in the intestines, which is conducive to intestinal health [60]. However, this study did not detect intestinal fermentation products (e.g., SCFAs); therefore, the association between alterations in *Romboutsia_ilealis* and *Faecalibacillus_intestinalis* and specific physiological functions cannot be determined. The relevant functional mechanisms require further verification by combining metabolomic data in subsequent studies. The results of the correlation analysis revealed that the effects of Paramylon on intestinal metabolites related to nutrient digestion and absorption were associated with a reduction in the relative abundance of *Lactobacillus_johnsonii* and an increase in that of *Romboutsia_ilealis*. This microbial shift may be involved in the observed promotion of amino acid and glucose metabolism, suggesting a potential link between the Paramylon-induced alterations in gut microbiota and host metabolic outcomes.

Notably, the distinct effects observed in this study may be intrinsically linked to the unique structural properties of Paramylon. Unlike cereal β-glucans, which are characterized by linear chains with mixed β-(1→3) and β-(1→4) linkages and high water solubility, Paramylon from *Euglena gracilis* possesses an highly linear β-(1→3)-linked backbone with negligible branching. This fundamental difference confers distinct physicochemical properties: Paramylon is insoluble in water and forms crystalline microfibers, while cereal β-glucans are soluble and form viscous solutions. Moreover, Paramylon typically exhibits a very high molecular weight (often >500 kDa) and a high degree of polymerization. We hypothesize that the beneficial effects observed are attributable to the unique structural properties of Paramylon. Its resistance to digestion likely allows it to function as a persistent prebiotic modulator and a specialized fermentation substrate for gut microbiota, thereby promoting host health. The precise mechanistic pathways warrant further investigation.

Therefore, our research results indicate that under restricted feeding conditions, adding Paramylon can effectively regulate the intestinal microbiota, thereby influencing the changes in related metabolites, and is beneficial to the intestinal digestion and absorption process as well as the intestinal health of piglets.

## 5. Conclusions

In conclusion, under restricted feeding conditions, 0.05% dietary Paramylon supplementation significantly improved nutrient digestibility and absorption in weaned piglets. These findings demonstrate Paramylon’s potential as an eco-friendly functional feed additive. The observed beneficial effects are likely mediated through multifaceted adaptations of the intestinal epithelium. These include enhanced digestive enzyme activity, upregulation of genes involved in nutrient digestion and transport, and a direct interaction of Paramylon with intestinal epithelial cells. Furthermore, the increase in intestinal beneficial bacteria may positively modulate the host’s metabolic profile, synergistically contributing to the overall improvement in nutrient utilization. However, it should be noted that the basal diet used in this study contained ZnO. Consequently, the beneficial effects of Paramylon observed herein occurred in the presence of ZnO. Future studies using ZnO-free diets are warranted to elucidate its standalone efficacy. Notably, our findings are specific to the critical immediate post-weaning period. Further research is warranted to determine whether the observed short-term benefits of Paramylon in weaned piglets translate into sustained improvements in growth performance and overall health status throughout the nursery and grow-finish phases. Furthermore, a more in-depth study of the interaction between Paramylon and other microbial metabolite products will be beneficial for its practical application in production.

## Figures and Tables

**Table 1:** Ingredient compositions and nutrient levels of the basal diet (as-fed basis).

Ingredient	Content (%)	
	CTRL	Paramylon
Corn	21.50	21.50
Expanded Corn	15.00	15.00
Broken Rice	15.00	15.00
Soybean Meal (43% CP)	10.00	10.00
Fermented Soybean Meal	7.60	7.60
Expanded Soybean	5.00	5.00
Low-Protein Whey Powder	7.50	7.50
Whole Milk Powder	5.00	5.00
Imported Fish Meal (CP 65%)	4.00	4.00
Coconut Oil Powder	0.50	0.50
Soybean Oil	0.50	0.50
Glucose	2.00	2.00
Sucrose	2.00	2.00
98 L-Lysine HCL	0.49	0.49
DL-Methionine	0.28	0.28
L-Threonine	0.25	0.25
L-Tryptophan	0.09	0.09
Valine	0.09	0.09
Choline Chloride (50%)	0.15	0.15
Limestone	0.50	0.50
Dicalcium Phosphate (17/21)	1.20	1.20
Sodium Chloride	0.40	0.40
Zinc Oxide	0.20	0.20
Compound Acid	0.5	0.5
Mineral Premix	0.20	0.20
Vitamin Premix	0.05	0.05
Total	100.00	100.00
Nutrient Levels		
Net Energy (Swine), Mcal/kg	2.65	2.65
Crude Protein, %	19.00	19.00
Calcium, %	0.80	0.80
STTD Phosphorus, %	0.37	0.37
SID Lysine, %	1.35	1.35
SID Methionine, %	0.57	0.57
SID Methionine + Cystine, %	0.81	0.81
SID Threonine, %	0.85	0.85
SID Tryptophan, %	0.28	0.28
SID Valine, %	0.89	0.89
SID Isoleucine, %	0.71	0.71

Paramylon was supplemented at each concentration as an additive. Its nutritional contribution was considered negligible and did not displace any basal ingredients. The mineral and vitamin premixes are provided per kilogram of diet: Fe, 100 mg; Cu, 6 mg; Zn, 100 mg; Mn, 4 mg; I, 0.14 mg; Se, 0.35 mg. VA, 12,000 IU; VD3, 2400 IU; VE, 100 mg; VK3, 4.8 mg; VB1, 2 mg; VB2, 7.2 mg; VB6, 3.6 mg; VB12, 0.03 mg; niacin, 40 mg; pantothenic acid, 25 mg; folic acid, 4 mg; biotin, 0.48 mg.

**Table 2:** Primers used for gene expression analysis by real-time PCR.

Gene	Forward Sequence (5′-3′)	Reverse Sequence (5′-3′)
SGLT1	CCACTTTCCCTATAAAACCTCAC	CTCCATCAAACTTCCATCCTCAG
SLC7A1	TCTGAACGTCCGTCAGTTCG	GCGTCTAGCAACCCGGC
SLC1A5	AGCAAGGAGGTGCTCGATTC	GAGTAGGACGTCGCGTATGAG
SLC6A19	AACCTCTTCTGGCAGGTCAC	TGGGAAACTCCTCATAGGCTG
SLC7A7	CAATGCCTCCATTGTGGCTG	AGCGCCATGAGACCATTGAA
PEPT1	CATCGCCATACCCTTCTG	TTCCCATCCATCGTGACATT
SLC38A2	GCTGTGGCTGTCATTGCTGT	TGGTAGCCGATGAGGATGAC
SLC2A2	ACCGGAATGTTCTTAGCCCC	GCTGTACGCAAAACCCGAAG
GLUT2	CCTGCTTGGTCTATCTGCTGTG	TTGATGCTTCTTCCCTTTCTTT
GLUT5	GACCGGCATCGCCATTTTC	CGATGCTGAGGTAGGGCATC
EAAT3	ATAGAAGTTGAAGACTGGGAAAT	GTGTTGCTGAACTGGAGGAG
FATP2	AACTACAACATCCGCGGGAA	GCTGCTTGTAGATCTGGCGA
FATP3	TGGAGACACCTTCAGGTGGAA	CTGGCACAGCGACTCCATAG
LAT1	CTGCTGGTCATCGTCTTCCT	CAGGTAGCCAGGGTCATAGC
FABP1	TTCATGAAGGCAGTCGGTCT	CACGATTTCCGATGTCCCCT
β-actin	CTACGCCAACACGGTGCTGTC	CTCCTGCTTGCTGATCCACATCTG

**Table 3:** Effects of Paramylon on growth performance in weaned piglets.

Item	Group				SEM	*p*-Value		
	CTRL	0.025% Paramylon	0.05% Paramylon	0.10% Paramylon		Treatment	Linear	Quadratic
Initial body weight, kg/pig	7.24	7.13	7.20	7.30	0.108	0.956	0.795	0.647
Final body weight, kg/pig	11.37	12.02	13.29	11.81	0.296	0.126	0.312	0.069
ADG, kg/d	0.17	0.18	0.22	0.16	0.008	0.060	0.693	0.038
ADFI, kg/d	0.40 ^b^	0.39 ^b^	0.46 ^a^	0.37 ^b^	0.010	0.007	0.982	0.030
F/G	2.49	2.27	2.21	2.41	0.097	0.749	0.763	0.303

ADG: average daily gain; ADFI: daily feed intake; F/G: feed conversion ratio. CTRL: basal diet, 0.025% Paramylon: basal diet supplemented with 0.025% Paramylon, 0.05% Paramylon: basal diet supplemented with 0.05% Paramylon, 0.10% Paramylon: basal diet supplemented with 0.10% Paramylon. Data are presented as the mean. SEM: standard error of the mean. ^a,b^ Means in the same row with different letters differ (*p* < 0.05).

**Table 4:** Effects of Paramylon on serum biochemical parameters in weaned piglets.

Item	Group				SEM	*p*-Value		
	CTRL	0.025% Paramylon	0.05% Paramylon	0.10% Paramylon		Treatment	Linear	Quadratic
TC, mmol/L	2.37 ^ab^	2.06 ^bc^	2.44 ^a^	2.01 ^c^	0.066	0.034	0.185	0.602
TG, mmol/L	0.48	0.61	0.55	0.54	0.034	0.609	0.753	0.323
Urea, mmol/L	4.12 ^a^	4.03 ^a^	3.31 ^b^	4.44 ^a^	0.130	0.011	0.800	0.010
LDL-C, mmol/L	1.19	1.04	1.13	1.03	0.033	0.286	0.212	0.691
HDL-C, mmol/L	0.89	0.92	1.01	0.87	0.039	0.322	0.453	0.114
TP, g/L	45.80	43.94	47.13	42.92	1.296	0.699	0.658	0.666
ALB, g/L	21.53	19.68	21.56	19.17	0.697	0.535	0.431	0.849
GLB, g/L	24.28	24.25	25.57	23.74	0.870	0.913	0.973	0.629
A/G, g/L	0.90	0.84	0.86	0.81	0.034	0.856	0.478	0.939

TC: total cholesterol; TG: total triglyceride; LDL-C: low-density lipoprotein cholesterol; HDL-C: high-density lipoprotein cholesterol; TP: total protein; ALB: albumin; GLB: globulin; A/G: albumin-to-globulin ratio. CTRL: basal diet, 0.025% Paramylon: basal diet supplemented with 0.025% Paramylon, 0.05% Paramylon: basal diet supplemented with 0.05% Paramylon, 0.10% Paramylon: basal diet supplemented with 0.10% Paramylon. Data are presented as the mean. SEM: standard error of the mean. ^a,b,c^ Means in the same row with different letters differ (*p* < 0.05).

**Table 5:** Effects of Paramylon on serum free amino acid concentrations in weaned piglets.

Item	Group		SEM	*p*-Value
	CTRL	0.05% Paramylon		
P-Ser, μg/mL	1.62	1.57	0.184	0.789
Tau, μg/mL	19.44	22.48	2.629	0.284
PEA, μg/mL	1.02	1.27	0.298	0.434
Urea, μg/mL	4.90	5.44	0.658	0.439
Asp, μg/mL	1.85	2.19	0.258	0.209
Thr, μg/mL	24.25	17.68	5.597	0.523
Ser, μg/mL	9.91	10.62	1.269	0.587
Glu, μg/mL	16.36	16.15	2.009	0.920
α-AAA, μg/mL	6.86	7.68	0.758	0.575
Gly, μg/mL	26.82	29.52	5.312	0.625
Ala, μg/mL	18.63	18.60	0.025	0.995
Cit, μg/mL	5.31	5.63	0.604	0.615
α-ABA, μg/mL	4.31	3.19	0.573	0.080
Val, μg/mL	15.17	14.82	0.356	0.714
Cys, μg/mL	4.74	4.87	0.518	0.808
Met, μg/mL	2.36	2.38	0.219	0.954
Cysthi, μg/mL	1.98	1.99	0.197	0.957
Ile, μg/mL	7.33	5.44	2.296	0.115
Leu, μg/mL	6.66	7.57	1.041	0.419
Tyr, μg/mL	4.14	3.92	0.661	0.741
Phe, μg/mL	5.55	5.60	0.693	0.943
β-Ala, μg/mL	0.89 ^a^	0.57 ^b^	0.098	0.012
Hylys, μg/mL	1.03	1.46	0.683	0.540
Orn, μg/mL	2.83	2.92	0.252	0.715
Lys, μg/mL	10.35	12.24	0.994	0.125
1Mehis, μg/mL	1.66	1.88	0.243	0.382
His, μg/mL	2.58	3.29	0.354	0.074
3Methis, μg/mL	0.86	0.80	0.204	0.796
Car, μg/mL	1.01 ^b^	2.54 ^a^	0.525	0.024
Arg, μg/mL	12.71	10.55	1.113	0.099

P-Ser: Phosphoserine; Tau: Taurine; PEA: Phosphoethanolamine; Asp: Aspartic Acid; Thr: Threonine; Ser: Serine; Glu: Glutamic Acid; Sar: Sarcosine; α-AAA: α-Aminoadipic Acid; Gly: Glycine; Ala: Alanine; Cit: Citrulline; α-ABA: α-Aminobutyric Acid; Val: Valine; Cys: Cystine; Met: Methionine; Cysthi: Cystathionine; Ile: Isoleucine; Leu: Leucine; Tyr: Tyrosine; Phe: Phenylalanine; β-Ala: β-Aminoisobutyric Acid; Hylys: Hydroxylysine; Orn: Ornithine; Lys: Lysine; 1Mehis: 1-Methylhistidine; His: Histidine; 3Methis: 3-Methylhistidine; Car: Carnosine; Arg: Arginine. CTRL: basal diet, 0.05% Paramylon: basal diet supplemented with 0.05% Paramylon. Data are presented as the mean. SEM: standard error of the mean. ^a,b^ Means in the same row with different letters differ (*p* < 0.05).

**Table 6:** Effects of Paramylon on the nutrient digestibility in weaned piglets.

Item	Group		SEM	*p*-Value
	CTRL	0.05% Paramylon		
Digestibility of nutrients, %				
Dry matter	76.40 ^b^	85.50 ^a^	1.224	0.001
Crude protein	61.12 ^b^	78.34 ^a^	5.975	0.028
Crude fat	72.44 ^b^	82.54 ^a^	1.629	<0.001
Crude fiber	76.71 ^b^	85.47 ^a^	1.076	<0.001
Gross energy	77.45 ^b^	85.70 ^a^	1.036	<0.001

CTRL: basal diet, 0.05% Paramylon: basal diet supplemented with 0.05% Paramylon. Data are presented as the mean. SEM: standard error of the mean. ^a,b^ Means in the same row with different letters differ (*p* < 0.05).

**Table 7:** Effects of Paramylon on intestinal morphology in the jejunum.

Item	Group		SEM	*p*-Value
	CTRL	0.05% Paramylon		
Villu height, μm	238.15 ^b^	268.22 ^a^	10.451	0.005
Crypt depth, μm	276.29 ^a^	248.19 ^b^	10.377	0.008
Villus height/crypt depth	0.90 ^b^	1.16 ^a^	0.064	<0.001

CTRL: basal diet, 0.05% Paramylon: basal diet supplemented with 0.05% Paramylon. Data are presented as the mean. SEM: standard error of the mean. ^a,b^ Means in the same row with different letters differ (*p* < 0.05).

**Table 8:** Effects of Paramylon on digestive enzymes in the ileum.

Item	Group		SEM	*p*-Value
	CTRL	0.05% Paramylon		
TRY, U/mg prot	4014.36	6897.56	1288.741	0.060
LPS, U/mL	0.62	0.72	0.167	0.562
β-AL, U/g	4.15 ^b^	5.75 ^a^	0.562	0.022

TRY: trypsin, LPS: lipase, β-AL: β-amylase. CTRL: basal diet, 0.05% Paramylon: basal diet supplemented with 0.05% Paramylon. Data are presented as the mean. SEM: standard error of the mean. ^a,b^ Means in the same row with different letters differ (*p* < 0.05).

**Table 9:** Effects of Paramylon on the relative abundance of the top 10 microorganisms at the phylum level in the ileum of weanling piglets.

Item	Group		SEM	*p*-Value
	CTRL	Paramylon		
Firmicutes, %	69.37	65.02	14.789	0.773
Proteobacteria, %	14.23	13.09	4.798	0.815
Actinobacteriota, %	6.88	4.12	3.239	0.409
Unassigned, %	3.60	0.94	2.526	0.326
Others, %	2.75	5.21	3.228	0.467
Unclassified_Bacteria, %	1.00	2.11	1.376	0.436
Bacteroidota, %	1.00	1.33	0.499	0.531
Chloroflexi, %	0.50	4.18	2.581	0.197
Acidobacteriota, %	0.50	2.39	1.444	0.231
Desulfobacterota, %	0.13	0.54	0.309	0.231

**Table 10:** Effects of Paramylon on the relative abundance of the top 10 microorganisms at the genus level in the ileum of weanling piglets.

Item	Group		SEM	*p*-Value
	CTRL	Paramylon		
Others, %	37.61	39.08	15.82	0.927
Clostridium_sensu_stricto_1, %	18.80	10.62	8.590	0.361
Streptococcus, %	15.28	3.43	6.313	0.099
Actinobacillus, %	6.91	4.25	4.574	0.497
Romboutsia, %	4.70 ^b^	32.24 ^a^	9.556	0.021
Turicibacter, %	5.14	2.74	2.487	0.358
Lactobacillus, %	4.15 ^a^	0.76 ^b^	1.137	0.017
Unassigned, %	3.60	0.94	2.526	0.326
Terrisporobacter, %	2.59	5.82	2.531	0.223
Limosilactobacillus, %	1.20	0.62	0.647	0.385

^a,b^ Means in the same row with different letters differ (*p* < 0.05).

## Data Availability

The data that support the findings of this study are available from the corresponding author upon reasonable request.
